# Addressing the rights and well‐being of domestic workers in Africa: Status in Malawi

**DOI:** 10.1002/puh2.123

**Published:** 2023-09-16

**Authors:** Symon Fidelis Nayupe, Alieu Touray, Dalitso Tembo, Mama Tamanda Msiska, Juliana Mputeni, Allan Hans Muhome

**Affiliations:** ^1^ University Private Clinic Kamuzu University of Health Sciences Blantyre Malawi; ^2^ Department of Sociology University of Kerala, Karyavattom Campus Thiruvananthapuram India; ^3^ Truth, Reconciliation and Reparations Commission Banjul The Gambia; ^4^ Catholic Commission for Justice and Peace Chikwawa Malawi; ^5^ Nursing Department Nkhotakota District Hospital Nkhotakota Malawi; ^6^ Community Health Department Partners in Health Neno Malawi; ^7^ Malawi Law Society Blantyre Malawi

**Keywords:** domestic worker, health, human rights, Malawi, well‐being

## Abstract

Domestic work continues to contribute to employment in most developing countries significantly. Over 75.6 million people are domestic workers worldwide, three‐quarters of whom are women. In Africa, many people still rely on domestic work for employment, making up 2.2% of the total workforce on the continent. With a predominant presence in the sub‐Saharan region, Africa's domestic workforce is estimated at around 9.6 million. Mirroring the global trend, most workers are women, with an estimated 15.8% of Africa's paid female employees being domestic workers. This considerable presence of domestic work contributes to domestic and intra‐continental migration in Africa, where about 80% of the domestic workers are from within. The Southern African region is a major intra‐continent destination for most workers. In Malawi, domestic labour is also common and is one of the country's primary sources of jobs, especially for poor populations who often domestically migrate from rural to urban areas. However, country estimates for the total number of domestic workers are yet to be published. Although there have been improved work conditions for domestic workers in some countries worldwide, the situation is different in many African nations, including Malawi. Domestic workers still face underpayment, long working hours, physical and psychological ill‐treatment from employers, challenging, harsh working environments and lack of access to healthcare. We discuss the status of the well‐being of domestic workers in Malawi, a country in southeastern Africa. We note that the working conditions for domestic workers continue to be unfavourable, with physical and psychological abuse and other human rights violations. To improve the well‐being and working conditions of domestic workers, government and non‐governmental organisations should, among other things, facilitate programmes to root out poverty, strengthen the legal framework that addresses domestic worker abuse and ensure social protection, including healthcare protection.

## INTRODUCTION

The International Labour Organisation (ILO) defines domestic workers as individuals providing household services, such as cleaning, gardening, taking care of children or newborn babies and older people and other household chores [1]. According to the ILO global statistics for 2021, about 75.6 million people are domestic workers worldwide, three‐quarters of whom are women. About 9.6 million workers are in Africa, most working in sub‐Saharan Africa [1]. As is the case globally, most domestic workers in Africa are females, with about 15.8% of the total number of paid female employees on the continent being domestic workers. It is evident that many people still rely on domestic work for employment on the continent, as domestic work makes up about 2.2% of the total workforce [1]. About 80% of the domestic workers are from within, with the Southern African region being a major intra‐continent destination. These workers are often from rural areas, migrating to urban areas for low‐skilled jobs [2, 3].

In Malawi, domestic labour is also common and is one of the country's primary sources of jobs, especially for poor populations. As a developing country with low income and a predominantly rural population, many individuals, particularly women and young children, resort to domestic work for the survival of their families [4, 5]. Although domestic work is common, country estimates for the total number of domestic workers are yet to be published. Only a few surveys in selected districts have been conducted, but even these still need to provide a total estimate for those districts [5, 6]. These surveys demonstrated that domestic work is common, and most domestic workers were women. Of additional note is the presence of child labour within the domestic workforce in Malawi. A 2018 report by ILO estimated that there were 2.1 million child labourers (aged 5–17 years) by that year, with most of them working on farms and in domestic work [7].

We discuss the current state of the work environment for domestic workers in Malawi and its impact on their social and health well‐being. We present recommendations on strategies to improve their work conditions and recognition as human beings who deserve decent work conditions and proper treatment as workers for good social and health well‐being.

## SOCIAL PROTECTION AND HUMAN RIGHTS OF DOMESTIC WORKERS

Human rights are the inherent freedoms and rights everyone has by being human. According to the Universal Declaration of Human Rights, ‘all human beings are born free and equal in dignity and rights’ irrespective of their social and economic status [8]. Social protection, on the other hand, is defined as ‘a set of policies and programs designed to reduce and prevent poverty and vulnerability during our lives’ [9]. To the domestic worker, social protection would include maternity protection, sickness benefits, health protection and disability benefits. Although domestic workers deserve these provisions as aspects of human rights, the ILO indicates that many domestic workers worldwide lack these provisions. Workers continue to be vulnerable to human rights abuses and denial of social protection by employers [1, 3, 9, 10].

As a continental background, in Africa, there are several issues underlying domestic worker abuse, even though most countries put legislation protecting and promoting the fundamental rights of all. The ILO highlighted various forms of violations domestic workers face in Africa, such as ‘the gender dimension, inappropriate legal coverage, statistics and data gaps, labour exploitation and social structures, migration of domestic workers, trafficking of children and women into domestic work, as well as high informal employment’ [2]. Poor social backgrounds and economic conditions force many who often have no other better alternatives into domestic work [1, 4]. This promotes symbolic violence from their employers, who have the more significant social and financial power. Despite their efforts in the households, most domestic workers’ services are undervalued and unprotected, making them vulnerable and victims of waves of abuse.

The situation for domestic workers in Malawi regarding human rights and social protection is no different from the findings of ILO and other labour and human rights organisations on the treatment of domestic workers in Africa [1, 2, 11]. Other studies on the work conditions for domestic workers in Malawi have been documented and have reported similar findings [4, 5, 6, 7]. With many women and children in the domestic labour workforce, Malawi's domestic worker human rights victims are primarily women and children. Nonetheless, it is essential to emphasise that all groups of domestic workers (females, children and males) in Malawi experience social protection challenges and human rights abuse [4, 6, 7].

One of the most prominent issues is low wages perpetrated by the decisional power gap between employers and domestic workers. Employers are on one end with near‐total power, whereas employees are on another end, almost entirely powerless. With this, workers have little say about their wages and provisions for social protection packages. For child workers, they have little or no bargaining power because they are too young to negotiate. With little law enforcement against such acts, women, men and children continue working, receiving meagre wages that hardly support a comfortable living [5, 12]. In these situations, it is unsurprising that many domestic workers in Malawi are paid less than the government‐set minimum wage of 38,000 Malawian Kwacha (about 35.38 US dollars) and additionally experience withholding of wages and salaries [4, 5, 13]. It is also common that most of them work more than the statutory 8 h a day for which they get no additional pay, subjecting them to what is termed contemporary slavery [5]. Unpublished records from Malawi's Industrial Relations Court indicate that most cases filed by domestic workers are based on unfair dismissal and complaints of withheld wages.

Furthermore, most domestic workers in Malawi would be described as experiencing structural violence. The working environment of domestic workers subjects them to situations where employers socially ostracise them out of hostility and suspicions of thievery. This significantly limits the workers’ social involvement in society and constrains them from meeting their basic needs and achieving the quality of life that would otherwise be possible [14]. The Malawi labour laws, specifically the Employment Act, outline general conditions for all employed individuals [15]. These conditions, however, only sometimes conform to the nature of domestic work. In most cases, employers hardly apply the legal requirements for the remuneration and treatment of domestic workers, thereby perpetrating the suffering of workers.

## SOCIAL AND HEALTH WELL‐BEING STATUS OF DOMESTIC WORKERS

The current framework for domestic workers in Malawi does not include health benefits. Workers rarely have medical insurance or some form of medical coverage. They must cover the costs of their health. Although this is the case, it is essential to highlight that most conditions in which domestic workers work do not promote healthy behaviours [5]. It is commonplace to have domestic workers work for extended hours, carry out exhausting or physically dangerous activities and not feed nutritiously to promote health [4, 5]. Domestic workers risk intoxication from cleaning substances and physical and emotional abuse from employers, leading to mental health problems, such as depression, psychosis and mood disorders [16]. These poor working conditions pose immediate risks to domestic workers and might put them at the risk of developing significant health complications even in the long term [17].

Although Malawi has a public healthcare system that citizens can access, some domestic workers still report being unable to access such health services as employers will not, at times, allow them to seek healthcare services when sick. These workers are accused of feigning illness to ‘run away from work’. For those who have the chance to access such services, it is noted that they are often left to seek those services alone, with some being too sick to do so on their own. Thus, the burden of the domestic worker is two‐edged: to work in unhealthy environments and then single‐handedly bear the responsibility when an illness develops.

## FACTORS IMPACTING DOMESTIC WORK IN MALAWI AND RECOMMENDATIONS

### Formalisation or professionalisation of domestic work

In Malawi and many other African countries, domestic work is informal. Many workers are in private homes or properties where they were recruited informally, either personally or by agents not recognised by law [1, 6]. The informal recruitment process disadvantages the entire domestic worker workforce due to the absence of legal references to procedures or guidelines that would protect workers. Additionally, part of the formalisation involves the establishment of a legal framework that recognises domestic work under a country's labour laws [18]. Malawi has previously amended its labour law to give provisions for guidance on domestic workers on various issues, such as minimum wages, gratuity and other terminal benefits, maximum working hours, and working conditions [19, 20]. However, although these legal provisions exist, many employers disregard them as they are not adequately enforced. Furthermore, access to legal redress is limited, often lengthy and costly for a domestic worker [21]. These gaps propel the mistreatment of workers as often there is no punishment for the perpetrators of domestic workers’ injustice. The government should, therefore, move to enforce legal provisions for domestic workers and adequately recognise workers and their access to legal services.

### Clear job descriptions for domestic workers

There needs to be a more apparent distinction on what is expected from a domestic worker. They are sometimes given roles beyond domestic work per ILO definition. Such roles may be quite involving and, at times, require some level of professionalism. For example, in many cases, a domestic worker must undertake the nanny or babysitter duties on top of the cleaning and catering services. The role of a babysitter is directly aligned with early childhood development. It is complex as it requires diverse skills in ‘knowing, being, experiencing, and acting’, a keen observer of children's needs, and a teacher of skills and knowledge in literacy and numeracy [22]. Lack of clear distinction between domestic workers’ roles and responsibilities is a recipe for massive exploitation and abuse. Domestic work should, therefore, have a description of duties that would outline the expectations and limits of services.

### Focus on women and child rights in domestic work

There is a substantial indication of abuse of domestic workers in the context of them being female. For example, there are cases where these female domestic workers are forced into sexual relationships or encounters with their male employers. The difficulty with the workers denying engaging in such acts is that they are often threatened with job termination or that some other serious accusation will be laid against them [4, 7].

When women domestic workers fall pregnant, there is sometimes no provision for maternity leave. Often, pregnancy results in the termination of their job. To maintain their desperately needed jobs, women domestic workers are denied exercising their sexual and reproductive health rights by the administration of such job termination threats [23]. The courts in Malawi have held that termination based on pregnancy is an unfair labour practice and ordered compensation to be paid [24]. However, the court processes are expensive for domestic workers and long, taking an average of 3 years to complete a case.

Regarding child labour, it is noted that although Malawi agrees with various international conventions on child labour and has a legal framework banning child labour in its labour laws, the country still faces child labour challenges [25]. In domestic work, one of the significant loopholes is the absence of legal guidance on the minimum age requirement for employment in private homes. Such a requirement only exists for other sectors, such as agriculture, even though this also needs to meet international standards [15, 26].

Furthermore, for many years since the ratification of international conventions on child labour, the government has yet to adequately fund the operations of agencies enforcing laws and implementing policies against child labour, making the execution of the fight against child labour inefficient [25, 27]. Nonetheless, efforts have been made to coordinate activities related to fighting child labour, such as putting national and district bodies that oversee matters about child labour, developing and implementing policies against child labour and introducing social programmes focused on child labour [25].

Addressing the gaps regarding child labour in Malawi's legal framework and enforcement of the legal provisions by increasing funding for activities against child labour, among others, are some of the steps the government needs to take to eliminate child labour.

### Literacy and domestic work

The Malawian domestic work landscape comprises individuals from rural areas who have yet to have formal education. Some joined domestic work as children, missing education at that level [3, 23, 25, 28]. The high illiteracy levels among domestic workers contribute to their need for a more understanding of their rights. As a result, predatory employers, who are often well educated, take this as an advantage to treat the workers in a manner pleasing to themselves, often violating the basic rights of these workers. Young domestic workers should be encouraged to go back to school, as has already been initiated by some organisations in Malawi [29]. Through such initiatives, children are rescued from labour and returned to class. Civic education on human rights is recommended for older domestic workers who may not have the chance for formal education.

### Economic strengthening interventions

As poverty has been cited as the primary contributor to domestic work, alleviating poverty through programmes that strengthen the economic independence of the people is one strategy to lessen the desperation in people. Alternative sources of jobs that ensure long‐term economic sustenance in poor populations could empower the people and remove them from the disadvantage of having one choice of survival (Table 1).

**TABLE 1:** Summary of the factors impacting domestic work in Malawi and recommendations.

Factor	Recommendation
The informal nature of domestic work and lack of professionalisation	Legal recognition of workers, make legal services accessible, enforce legal provisions Professionalisation of domestic work
Unclear or lack of job description for Domestic Workers	Clear job description for domestic workers
Women and child rights	Improve access to legal redress for gender‐based abuse Prohibition of child labour and fighting against existing child labour practices
Economic challenges	Strengthen the economic independence of the people Economic empowerment through alternative jobs
Illiteracy	Emphasis on education for school‐going children Civic education for adult workers for human rights awareness

## CONCLUSION

Domestic work contributes significantly to employment in Malawi. However, most workers suffer challenges, such as poor remunerations, physical or mental ill‐treatment and restrictive working environments. Additionally, there is no social protection framework for these workers despite continuing to work under challenging conditions, often risking their physical and mental well‐being. Successful legal redress for domestic workers is also hard to access for many domestic workers. There should be interventions from government and non‐governmental organisations by formalising domestic work, ensuring legal recognition of domestic workers and enforcement of legal provisions for domestic workers. Furthermore, a functional social protection framework that includes access to healthcare, terminal benefits and other benefits should be in place.

## AUTHOR CONTRIBUTIONS

*Conceptualisation; formal analysis; investigation; project administration; supervision; writing – original draft; writing – review and editing*: Symon Fidelis Nayupe. *Conceptualisation; formal analysis; writing – original draft; writing – review and editing*: Alieu Touray. *Conceptualisation; formal analysis; investigation; resources; writing – original draft; writing – review and editing*: Dalitso Tembo. *Conceptualisation; formal analysis; resources; writing – original draft; writing – review and editing*: Mama Tamanda Msiska and Juliana Mputeni. *Conceptualisation; formal analysis; investigation; project administration; resources; supervision; writing – original draft; writing – review and editing*: Allan Hans Muhome.

## CONFLICT OF INTEREST STATEMENT

The authors declare no conflicts of interest to any party.

## FUNDING INFORMATION

This did not receive any funding from any institution or organisation.

## Data Availability

None.
